# Venous thromboembolism in hospitalized patients receiving chemotherapy for malignancies at Japanese community hospital: prospective observational study

**DOI:** 10.1186/s12885-017-3326-1

**Published:** 2017-05-19

**Authors:** Hiromitsu Kitayama, Tomohiro Kondo, Junko Sugiyama, Kazutomo Kurimoto, Yasuhiro Nishino, Michiaki Hirayama, Yasushi Tsuji

**Affiliations:** 1 Department of Medical Oncology, Tonan Hospital, Kita 4 Nishi 7 3-8, Chuo-ku, Sapporo, Hokkaido 060-0004 Japan; 2 Department of Clinical Laboratory, Tonan Hospital, Kita 4 Nishi 7 3-8, Chuo-ku, Sapporo, Hokkaido 060-0004 Japan; 3 Department of Cardiovascular Medicine, Tonan Hospital, Kita 4 Nishi 7 3-8, Chuo-ku, Sapporo, Hokkaido 060-0004 Japan; 4 Department of Gastroenterology, Tonan Hospital, Kita 4 Nishi 7 3-8, Chuo-ku, Sapporo, Hokkaido 060-0004 Japan

**Keywords:** Antineoplastic agents, Antithrombin III-protease complex, Biomarkers, Blood coagulation, Fibrinolysis, Patient admission, Plasmin-plasmin inhibitor complex, Prospective studies, Risk factors, Venous thromboembolism

## Abstract

**Background:**

Although Asian population was recognized to have a lower risk of venous thromboembolism (VTE), its increasing prevalence and incidence remain unclear in patients with malignancies. We attempted to predict VTE development using activation markers of coagulation and fibrinolysis.

**Methods:**

We enrolled patients with malignancy admitted to Tonan Hospital between April and December 2014 to receive a new-for-them chemotherapy regimen. All patients were examined for VTE by computed tomography and whole-leg compression ultrasonography before chemotherapy and three months later. We also examined plasma levels of thrombin-antithrombin complex (TAT) and plasmin α2-plasmin inhibitor complex (PIC) before chemotherapy. The cut off values of TAT and PIC were set at 2.1 ng/mL and 1.8 μg/mL, respectively.

**Results:**

Of 97 patients, the majority (67%) had distant metastases. The most common malignancies were colorectal (26%), breast (23%), and stomach (19%) cancer. VTE was detected in 29 patients (31%); all were asymptomatic. VTE was newly developed in 12 patients in the three-month observation period, which means the incidence was 49 per 1000 person-years. Non-increased PIC with increased TAT was the only significant risk factor for both VTE prevalence and incidence in multivariate analysis, and the odds ratios were 3.0 (95% confidence interval, 1.1–8.2; *P* = 0.034) and 9.4 (95% confidence interval, 1.7–51.9; *P* = 0.011), respectively.

**Conclusions:**

The prevalence and incidence of VTE were high in hospitalized Japanese patients receiving chemotherapy for malignancies. Non-increased PIC with increased levels of TAT may be an independent risk factor for VTE development.

## Background

Cancer causes hypercoagulable states and thromboembolism [1]. No less than 11% of Western cancer patients develop thromboembolism, which is a secondary cause of cancer death [2, 3]. Hospitalization and chemotherapy are important risk factors for venous thromboembolism (VTE) [1, 4]. Prophylactic anticoagulation is generally recommended to hospitalized Western cancer patients [5, 6].

So far even VTE prevalence in Japanese cancer patients remains undetermined. Asian people have been recognized to have lower risk for VTE [7], however, estimated VTE incidence is definitely increasing in Japan [8]. It also remains unclear whether Asian cancer patients should receive prophylactic anticoagulation.

Increased plasma levels of coagulation activation markers, D-dimer and prothrombin fragment 1 + 2 (F1 + 2), were reported to be useful in prediction of VTE development [9–12]. Thrombin-antithrombin complex (TAT) reflects hypercoagulable states directly as with F1 + 2, and plasmin α2-plasmin inhibitor complex (PIC) reflects hyperfibrinolytic states [13]. What are the prevalence and incidence of VTE in hospitalized Japanese patients receiving chemotherapy? What are the biomarkers predicting VTE development? The purpose of this study is to investigate VTE rates prospectively and to predict the development using activation markers of coagulation and fibrinolysis.

## Methods

### Study population

We enrolled patients with histologically or cytologically confirmed malignancies admitted to Tonan Hospital, Sapporo, Japan, between April and December 2014 to receive a new-for-them chemotherapy regimen. Patients were required to have an acceptable hematologic, hepatic, and renal function for chemotherapy, and adequate performance status. Exclusion criteria included symptomatic thromboembolism, prophylactic anticoagulation, active infection, and pregnancy. Patients with asymptomatic VTE without anticoagulation diagnosed at enrollment were eligible in this study.

### Study protocol

All patients received inpatient chemotherapy. We prospectively observed the patients for three months. The main endpoint was an objectively confirmed VTE. We also collected following data: age, sex, body mass index, mobility, previous chemotherapy or hormone therapy, time from tumor onset, central venous access device, using anti-vascular endothelial cell growth factor antibody, recent trauma, surgery, or radiation therapy, complications including acute infection, tumor subtype, distant metastases, and laboratory data.

### Diagnostic imaging

All patients were examined for VTE by chest-abdomen-pelvic computed tomography (CT) and whole-leg compression ultrasonography before chemotherapy and three months later. The following veins were examined by a high frequency liner transducer of an Aplio XG SSA-790A ultrasound device (Toshiba Medical Systems, Toshiba Medical Systems Co., Ltd., Otawara, Japan): femoral, popliteal, and posterior tibial vein. Deep vein thrombosis (DVT) was diagnosed using compression maneuver and Doppler ultrasound technique, if the vein was non-compressible and blood flow compromised.

VTE was also diagnosed by direct visualization of a thrombus in CT scans of blood vessels. Arterial-phase scans covering pulmonary arteries were not performed for patients without PE symptoms. Contrast enhanced CT was also not performed in patient without adequate renal function, which means estimated glomerular filtration rate by Japanese equation is 45 mL/min/1.73 m^2^ or over [14].

### Laboratory data

Only at baseline, we measured CBC and serum levels of activation markers of coagulation and fibrinolysis: D-dimer, TAT, and PIC. D-dimer and PIC were measured by latex immunoassay kits: LIAS AUTO D-dimer NEO and LIAS AUTO PIC, respectively (Sysmex Co., Ltd., Kobe, Japan). TAT was determined by a chemiluminescent enzyme immunoassay kit: STACIA CLEIA TAT (LSI Medience Co., Ltd., Tokyo, Japan). The cut off values of each CBC and D-dimer were set based on Khorana and Vienna VTE risk assessment score, validated models to estimate the risk in Western patients receiving chemotherapy [15, 16]. Those of TAT and PIC were at 2.1 ng/mL and 1.8 μg/mL, 50th and 75th percentile of our data, respectively.

### Statistical analysis

This study was conducted as a preliminary assessment of VTE prevalence, incidence, and its risk factors. Continuous variables were expressed as mean ± standard division, mean ± standard error, or median (interquartile range), as appropriate, which were categorized because linearity on the logit scale could not be achieved with all continuous covariates. Odds ratio (OR) with 95% confidence interval (CI), calculated using Woolf’s method, is presented as risk ratio of VTE prevalence and incidence between groups. OR with the CI were calculated using Woolf-Haldane correction when an observed frequency has a value of zero. The difference in VTE prevalence and incidence between groups was analyzed with chi-square test. Pearson’s test with Yates’s continuity correction was used when expected frequencies were all over 5, otherwise data were analyzed with Fisher’s exact test. We used multivariate logistic regression model to confirm the interaction between TAT and PIC for VTE prevalence. The predictor variables were increased TAT, non-increased PIC, and the first-order interaction term (increased TAT × non-increased PIC). Selected risk factors for VTE prevalence and incidence were analyzed by the multivariate model. We assumed that TAT, PIC, or the combination would affect VTE prevalence and incidence. Therefore, these three factors and other factors which were *P* < 0.10 in the univariate analysis were selected for the multivariate analysis. TAT and PIC were analyzed separately from the combination to avoid multicollinearity by the multivariable analysis. Multicollinearity was assessed by using the variance inflation factor, which greater than 4.0 may be a cause for concern. All statistical tests were two-sided, and *P* < 0.05 was considered statically significant. All analyses were performed using EZR (Saitama Medical Center, Jichi Medical University, Saitama, Japan), which is a graphical user interface for R (The R Foundation for Stastical Computing, Vienna, Austria) [17]. More precisely, it is a modified version of R commander designed to add Stastical functions frequently used in biostatistics.

## Results

### Patient characteristics

A total of 99 patients were enrolled, of which 2 patients could not be followed up. Remaining 97 patients were observed for the three-month period. Table 1 summarizes baseline clinical characteristics available for analysis. All patients were Japanese with practically equal male-female distribution. Most patients had a central venous access device and did not require bedrest for three days. Part of the patients (18%) was treated with anti-vascular endothelial cell growth factor antibody. Few patients (9%) had a history of surgery or radiation therapy within one month. Practically no patients had obesity, chronic heart failure, rheumatic arthritis, or acute infection. No patients had a previous history of coagulation defects, VTE, chronic respiratory failure, nephrosis syndrome, or hormone therapy including erythropoietin. The majority (67%) had distant metastases, and a few patients (20%) had a history of chemotherapy. Time from tumor onset as well as specific tumor subtypes varied widely: the median was three months, and the majority subtypes (68%) were colorectal, breast, and stomach cancer.

**Table 1:** Baseline clinical characteristics (*n* = 97)

Characteristic	No. of patients	(%)
Age (yr.)	65	±12.1^a^
Sex ratio (M:F)	47	:50
Body mass index (kg/m^2^)	22	± 3.4^a^
Reduced mobility^c^	10	10
Previous chemotherapy	19	20
Time from tumor onset (yr.)	3	(1–12)^b^
Using anti-VGEF antibody	17	18
Recent (≤1 month) surgery	6	6
Recent (≤1 month) radiation	3	3
Complication		
Rheumatic arthritis	3	3
Chronic heart failure	1	1
Tumor type		
Digestive cancer		
Colon or rectum	26	27
Stomach	18	19
Esophagus	6	6
Pancreas	7	7
Other^d^	2	2
Female reproductive system cancer		
Breast	22	23
Ovary	3	3
Other tumor		
Lung cancer	2	2
Urinary tract cancer	2	2
Urachus cancer	1	1
Cancer of unknown primary	4	4
Sarcoma^e^	3	3
Lymphoma	1	1
Distant metastases	65	67

*VEGF* vascular endothelial cell growth factor

^a^Mean ± standard deviation. ^b^Median (interquartile range). ^c^Bedrest with bathroom privileges for at least three days, either due to patient’s limitation or on physician’s order. ^d^Liver and biliary tract. ^e^thymus and prostate.

### VTE

**Fig. 1:**
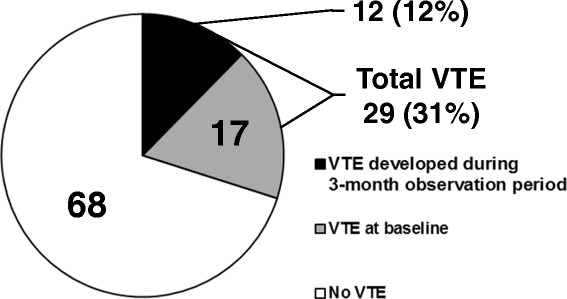
VTE prevalence in hospitalized patients receiving chemotherapy for malignancy (*n* = 97). VTE, venous thromboembolism; PE, pulmonary embolism

**Fig. 2:**
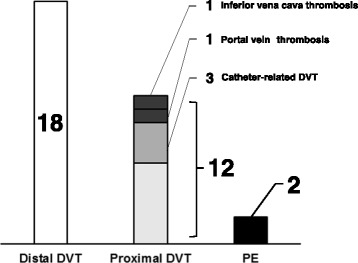
Details of total VTE (*n* = 29). Abbreviations are as in Fig. 1. There is some overlap. No deep vein thrombosis was found in upper-extremities

Figure 1 shows prevalence of VTE, which was detected in 29 patients (31%), 12 male and 17 female. VTE of 17 patients was detected before chemotherapy, and 12 (12%), 4 male and 8 female, were developed in the three-month observation period. VTE incidence was then 49 per 1000 person-years. Figure 2 shows VTE details of the 29 patients: 18 of which had distal DVT, 12 had proximal DVT, and 2 had PE. Three patients had both proximal and distal DVT, one had both distal DVT and PE, and one had both proximal DVT and PE. Almost all distal DVT was detected in soleus vein. Of the 12 proximal DVT, 9 were in lower extremity: iliac, femoral, or popliteal vein, 3 were catheter-related, 1 was in internal jugular vein, 1 in inferior vena cava, and 1 in portal vein. All VTE was asymptomatic, and only patients with proximal DVT and/or PE received anticoagulant therapy except where contraindicated or with limited life expectancy. One patient with proximal DVT was inserted inferior vena cava filter. No DVT progressed in size or number, and no patients suffered bleeding due to anticoagulation therapy.

### Predictor analysis of VTE prevalence

**Fig. 3:**
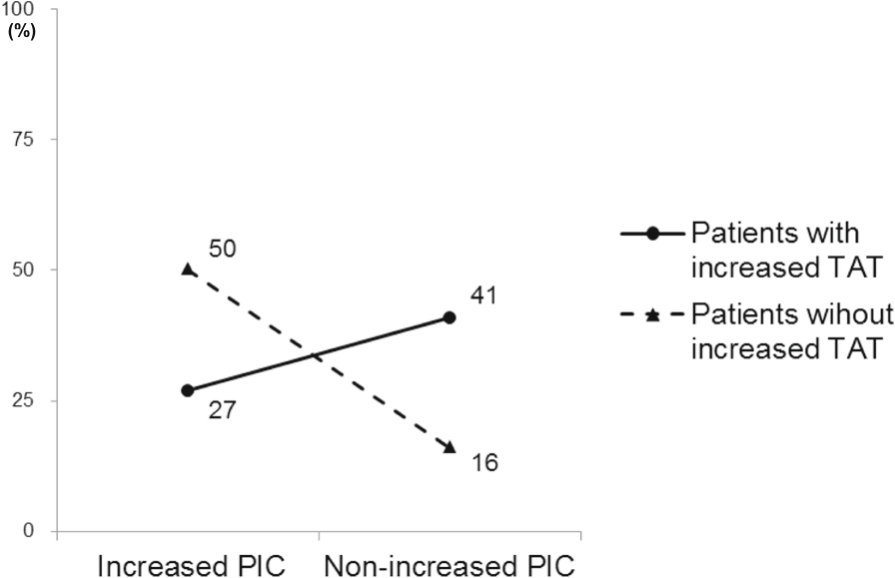
Change of VTE prevalence by non-increased PIC level in patients with and without increased TAT level. VTE, venous thromboembolism; TAT, thrombin-antithrombin complex; PIC, plasmin α2-plasmin inhibitor complex. Two crossing line graphs suggest interaction between increased TAT and non-increased PIC for VTE prevalence, which was significant (*P* = 0.043) Only in patients with increased TAT, non-increased PIC affects high prevalence of VTE

Table 2 shows univariate OR of risk factors for VTE prevalence, which was significantly higher in patients with reduced mobility (OR, 6.9; 95% CI, 1.6–29.0; *P* = 0.007). Elderly (≥70 years old) female was not a significant risk factor compared to male and female <70 years old, but lower limit of 95% CI of the OR was over 1.0 (3.7; 95% CI, 1.1–12.2; *P* = 0.054). Other factors were not significant risks. Figure 3 shows interaction between increased TAT and non-increased PIC, which was significant. VTE prevalence in patients with non-increased PIC was high (41%) with increased TAT levels, but low (16%) in patients without increased TAT. Lower limit of 95% CI of the OR was almost over 1.0 in a combination of non-increased PIC with increased TAT (OR, 2.6; 95% CI, 1.0–6.6; *P* = 0.082) by univariate analysis. Table 3 shows VTE prevalence of a high- and low-risk group based on tumor subtypes [6, 18]. The absolute rates of the high- and low-risk group were 38% and 25%, respectively, which is not a significant difference.

**Table 2:** Univariable analysis of risk factors for total VTE of 29 patients

Variable	No. of patients		(%)	OR	(95% CI)	*P*
Sex and age (yr.)						
< 70	17	/64	27	1.0	(ref.)	
≥ 70 male	4	/19	21	0.7	(0.2–2.5)	0.77
≥ 70 female	8	/14	57	3.7	(1.1–12.2)^a^	0.054
BMI						
< 25	21	/74	28	1.0	(ref.)	
≥ 25	8	/23	35	1.4	(0.5–3.6)	0.75
Reduced mobility^b^						
No	22	/87	25	1.0	(ref.)	
Yes	7	/10	70	6.9	(1.6–29.0)^a^	0.007^*^
Previous chemotherapy						
No	24	/78	31	1.0	(ref.)	
Yes	5	/19	26	0.8	(0.3–2.5)	0.92
Time from tumor onset (mo.)						
< 6 or >12	23	/85	27	1.0	(ref.)	
6–12	6	/12	50	2.7	(0.8–9.2)	0.17
Central venous access device						
No	1	/ 7	14	1.0	(ref.)	
Yes	28	/90	31	2.7	(0.3–23.6)	0.67
Using anti-VEGF antibody						
No	26	/80	33	1.0	(ref.)	
Yes	3	/17	18	0.5	(0.1–1.7)	0.26
Distant metastases						
No	7	/32	22	1.0	(ref.)	
Yes	22	/65	34	1.8	(0.7–4.9)	0.33
Developing acute infection^c^						
No	18	/67	27	1.0	(ref.)	
Yes	11	/30	37	1.6	(0.6–3.9)	0.46
Platelet count (/μL)^d^						
< 350,000	25	/87	29	1.0	(ref.)	
≥ 350,000	4	/10	40	1.7	(0.4–6.4)	0.48
Hemoglobin (g/dL)^d^						
≥ 10	26	/84	31	1.0	(ref.)	
< 10	3	/13	23	0.7	(0.2–2.6)	0.75
Leukocyte count (/μL)^d^						
< 11,000	29	/93	31	1.0	(ref.)	
≥ 11,000	0	/ 4	0	0.2	(0.0–4.7)	0.31
D-dimer (μg/mL)^de^						
< 1.5	7	/36	19	1.0	(ref.)	
≥ 1.5	19	/58	33	2.0	(0.8–5.4)	0.24
TAT (ng/mL)^de^						
< 2.1	10	/50	20	1.0	(ref.)	
≥ 2.1	16	/44	36	2.3	(0.9–5.8)	0.12
PIC (μg/mL)^d, e^						
≥ 1.8	7	/21	33	1.0	(ref.)	
< 1.8	19	/73	26	0.7	(0.2–2.0)	0.70
TAT ≥2.1 ng/mL and PIC <1.8 μg/mL^d, e^						
No	14	/65	22	1.0	(ref.)	
Yes	12	/29	41	2.6	(1.0–6.6)	0.082

*VTE* venous thromboembolism, *OR* odds ratio, *CI* confidence interval, *BMI* body mass index, *VEGF* vascular endothelial cell growth factor, *TAT* thrombin-antithrombin complex, *PIC* plasmin α2-plasmin inhibitor complex

^*^ *P* < 0.05. ^a^95% CI over 1.0. ^b^Bedrest with bathroom privileges for at least three days at baseline, either due to patient’s limitation or on physician’s order. ^c^During the three-month observation period. ^d^Measured at baseline, just before the first cycle of chemotherapy. ^e^Unmeasured in three patients

**Table 3:** Number of patients with total VTE according to underlying specific tumor subtypes

	Total VTE	No. of patients	Prevalence
Tumor type	(*n* = 29)	(*n* = 97)	(%)
High-risk group for VTE			
Stomach^a^	5	18	
Pancreas^a^	3	7	
Ovary^a^	1	3	
Lung^a^	2	2	
Urinary tract^a^	1	2	
CUP^b^	1	4	
Sarcoma^b^	2	3	
Lymphoma^a^	0	1	
Total	15	40	38^c^
Low-risk group for VTE			
Colon or rectum	6	26	
Breast	5	22	
Esophagus	3	6	
Biliary tract	0	1	
Liver	0	1	
Urachus	0	1	
Total	14	57	25

*VTE* venous thromboembolism, *CUP* cancer of unknown primary

^a^Listed as a risk factor for VTE in National Comprehensive Cancer Network Guidelines 2016 [6]. ^b^Listed as a risk factor for VTE in Asian patients [18]. ^c^Odds ratio was 1.84 vs. low-risk group for VTE (95% confidence interval, 0.8–4.4; *P* = 0.25).

Left side of Table 4 shows the multivariate OR of selected five risk factors. Non-increased PIC with increased TAT was the only significant risk factor (OR, 3.2; 95% CI, 1.1–8.2; *P* = 0.034). Lower limit of 95% CI of the OR was almost over 1.0 in elderly female (OR, 3.3; 95% CI, 0.9–12.4; *P* = 0.066), reduced mobility (OR, 4.9; 95% CI, 1.0–24.9; *P* = 0.057), and increased TAT (OR, 2.4; 95% CI, 0.9–6.4; *P* = 0.092). No evidence of multicollinearity between the five factors was found.

**Table 4:** Multivariable analysis of risk factors for total VTE of 29 patients and newly developed VTE of 12 patients (*n* = 97)

	Total VTE			Newly developed VTE		
Variable	OR	(95% CI)	*P*	OR	(95% CI)	*P*
Elderly female^a^	3.3	(0.9–12.4)	0.066		-	
BMI ≥25^b^		-		2.5	(0.5–12.0)	0.25
Reduced mobility^c^	4.9	(1.0–24.9)	0.057		-	
6–12 months from tumor onset^d^		-		3.0	(0.6–15.8)	0.19
Increased TAT^e^	2.4	(0.9–6.4)^f^	0.092	3.8	(0.7–20.8)^g^	0.12
Non-increased PIC^h^	1.2	(0.3–4.0)	0.78		-	
Increased TAT^e^ and non-increased PIC^h^	3.0	(1.1–8.2)^i^	0.034^*^	9.4	(1.7–51.9)^j^	0.011^*^

Abbreviations are as in Table 2. *VIF* variance inflation factor

^*^ *P* < 0.05. ^a^ ≥ 70 yr. vs. male and female <70 yr., adjusted for reduced mobility and non-increased PIC with increased TAT (VIF, 1.0). ^b^Adjusted for 6–12 months from tumor onset, and non-increased PIC with increased TAT (VIF, 1.0). ^c^Adjusted for elderly female and non-increased PIC with increased TAT (VIF, 1.0). ^d^Adjusted for BMI ≥25 and non-increased PIC with increased TAT (VIF, 1.1). ^e^ ≥ 2.1 ng/mL. ^f^Adjusted for elderly female and non-increased PIC (VIF, 1.1). ^g^Adjusted for BMI ≥25 and 6–12 months from tumor onset (VIF, 1.1). ^h^ < 1.8 ng/mL, adjusted for elderly female and increased TAT (VIF, 1.2). ^i^Adjusted for elderly female and reduced mobility (VIF, 1.0). ^j^Adjusted for BMI ≥25 and 6–12 months from tumor onset (VIF, 1.1)

### Predictor analysis of VTE incidence

Table 5 shows univariate OR of risk factors for VTE incidence in the three-month observation period, which was significantly higher in patients with high body mass index (OR, 4.0; 95% CI, 1.2–14.0; *P* = 0.030), 6–12 months from tumor onset (OR, 8.0; 95% CI, 2.0–31.8; *P* = 0.006), increased TAT (OR, 5.3; 95% CI, 1.1–26.6; *P* = 0.042), and non-increased PIC with increased TAT (OR, 12.0; 95% CI, 2.4–61.0; *P* = 0.001). Other factors did not show significantly higher OR.

**Table 5:** Univariable analysis of risk factors for newly developed VTE of 12 patients in three-month observation period

Variable	No. of patients		% per three months	OR	(95% CI)	*P*
Sex and age (yr.)						
< 70	7	/64	11	1.0	(ref.)	
≥ 70 male	1	/19	5	0.5	(0.05–3.9)	0.67
≥ 70 female	4	/14	29	3.3	(0.80–13.2)	0.10
BMI						
< 25	6	/74	8	1.0	(ref.)	
≥ 25	6	/23	26	4.0	(1.2–14.0)^a^	0.030^*^
Reduced mobility^b^						
No	11	/87	13	1.0	(ref.)	
Yes	1	/10	10	0.8	(0.1–6.7)	1.00
Previous chemotherapy						
No	9	/78	12	1.0	(ref.)	
Yes	3	/19	16	1.4	(0.4–5.9)	0.70
Time from cancer onset (mo.)						
< 6 or >12	7	/85	8	1.0	(ref.)	
6–12	5	/12	42	8.0	(2.0–31.8)^a^	0.006^*^
Central venous access device						
No	0	/7	0	1.0	(ref.)	
Yes	12	/90	13	2.4	(0.1–44.5)	0.59
Using anti-VEGF antibody						
No	11	/80	14	1.0	(ref.)	
Yes	1	/17	6	0.4	(0.1–3.3)	0.69
Distant metastases						
No	3	/32	9	1.0	(ref.)	
Yes	9	/65	14	1.6	(0.4–6.2)	0.75
Developing acute infection^c^						
No	24	/67	56	1.0	(ref.)	
Yes	5	/30	20	0.4	(0.1–1.1)	0.10
Platelet count (/μL)^d^						
< 350,000	12	/87	12	1.0	(ref.)	
≥ 350,000	0	/10	0	0.3	(0.0–5.2)	0.60
Hemoglobin (g/dL)^d^						
≥ 10	10	/84	12	1.0	(ref.)	
< 10	2	/13	15	1.4	(0.3–7.0)	0.66
Leukocyte count (/μL)^d^						
< 11,000	12	/93	13	1.0	(ref.)	
≥11,000	0	/4	0	0.7	(0.0–14.3)	1.00
D-dimer (μg/mL)^d, e^						
<1.5	3	/36	8	1.0	(ref.)	
≥1.5	7	/58	12	1.5	(0.4–6.3)	0.74
TAT (ng/mL)^d, e^						
< 2.1	2	/50	2	1.0	(ref.)	
≥ 2.1	8	/44	18	5.3	(1.1–26.6)^a^	0.042^*^
PIC (μg/mL)^d, e^						
≥ 1.8	0	/21	0	1.0	(ref.)	
< 1.8	10	/73	14	7.1	(0.4–126.5)	0.11
TAT ≥2.1 ng/mL and PIC <1.8 μg/mL^d, e^						
No	2	/65	3	1.0	(ref.)	
Yes	8	/29	28	12.0	(2.4–61.0)^a^	0.001^*^

Abbreviations and footnotes are as in Table 2.

Right side of Table 4 shows the multivariate OR of selected four risk factors. Non-increased PIC with increased TAT was the only significant risk factor (OR, 9.4; 95% CI, 1.7–51.9; *P* = 0.011), which had a good characteristic for predicting VTE development, 80% (8/10) of sensitivity with 75% (63/84) of specificity. No evidence of multicollinearity between the four factors was found.

## Discussion

In this study, we made two important clinical observations. First, the prevalence and incidence of VTE were high in hospitalized Japanese patients receiving chemotherapy for malignancies. The overall prevalence was 31%, high compared to that in Western countries. The prevalence of thromboembolism in Western cancer patients was reported to be about 11% [2, 3]. As for with VTE incidence, it was 49 per 1000 person-years in our study, also high compared to Western countries. In a meta-analysis, VTE incidence was estimated to be 13 (95% CI, 7–23) and 68 (95% CI, 48–96) per 1000 person-years among Western cancer patients at average and high risk for VTE, respectively [19]. VTE incidence in our study, 49 per 1000 person-years, was also high compared to Asian countries. From a nation-wide analysis in Taiwan, VTE incidence in hospitalized cancer patients was 1.9 per 1000 person-years [18]. In Japan, though from a questionnaire survey, estimated VTE incidence was 0.18 per 1000 person-years in 2006 [20]. These incidence rates of both Taiwan and Japan can be considered similar to relative risks of cancer and hospitalization in the Western counties, which was 4.7 (hazard ratio) and 2.63 (OR), respectively [4, 21]. Even considering these relative risks, VTE incidence in our study was high and equivalent to that of average- or high-risk for VTE in Western countries.

One important thing is that all patients in this study underwent whole-leg compression ultrasonography before and after inpatient chemotherapy. As far as we could find, no other study includes the ultrasonography for each hospitalized patient receiving chemotherapy for malignancy. This is one of the reasons why we observed such a lot of VTE patients. Hospitalized cancer patients receiving chemotherapy in Western countries must have more asymptomatic VTE than in Asia. The prevalence and incidence have been definitely underestimated. Despite the frequency, the natural history of asymptomatic distal DVT and their real risk of thromboembolic complications are still uncertain because of the scarcity of prospective, blind, nonintervention studies. In fact, no distal DVT in this study progressed after three months from inpatient chemotherapy without anticoagulation. Therefore it is still debated whether we should make the diagnosis and treatment. However, prophylactic anticoagulation in cancer patients may prevent symptomatic VTE, which is associated with a substantial risk of bleeding [22, 23]. For beneficial thromboprophylaxis, we need to define high-VTE risks.

The second clinical observation is that non-increased PIC with increased TAT may be an independent risk factor for VTE development. In this study, non-increased PIC with increased TAT was the only significant risk factor of both VTE prevalence and incidence. This is the first study to show the potential of the combination of coagulation and fibrinolytic activation markers to predict VTE development. Antithrombin III inactivates thrombin, which results in increased TAT levels and shows coagulation activation. α2-plasmin inhibitor inactivates plasmin, resulting in increased PIC levels, which shows fibrinolytic activation. In fact, other coagulation activation markers, F1 + 2 and D-dimer, were reported to be able to predict VTE [9–12]. Increased levels of F1 + 2 predicted a twofold increased risk of VTE in cancer patients [9]. Several studies indicate that D-dimer is associated with VTE risk in cancer patients [9–12], and the highest relative risk for VTE development (hazard ratio, 3.6) was found in patients with both increased F1 + 2 and D-dimer [9]. F1 + 2 and D-dimer are both coagulation activation markers, but D-dimer levels are also affected by fibrinolytic activation; D-dimer can be produced more in patients with pleural effusion, ascites, hematoma, or cancer itself. False-positive of D-dimer elevation for VTE is increased in patients with cancer patients. In disseminated intravascular coagulation, D-dimer levels are higher with advanced fibrinolysis than with suppressed fibrinolysis [24, 25]. F1 + 2 and D-dimer then complement each other in the identification of high-risk patients for VTE. In theory, the combination of TAT and PIC shows more simply and directly each activity of coagulation and fibrinolysis, respectively; non-increased PIC with increased TAT shows advanced coagulation with suppressed fibrinolysis, which means a state at high risk for thrombosis. False-positive for VTE can be decreased by the combination of TAT and PIC. According to our study, the interaction between increased TAT and non-increased PIC is also indicative of VTE related processes. Non-increased PIC with increased TAT might be a precise biomarker for VTE in patients with malignancies.

This study has two limitations. First, the number of VTE events was insufficient to detect differences in patients with and without each variable in multivariate analysis. The number of newly developed VTE events was only 12. This is a preliminary study to assess VTE prevalence, incidence, and its high-risk factors. Non-increased PIC with increased TAT was not adjusted for a number of VTE risk factors, except two: elderly female and reduced mobility. Second limitation is that blood sampling difficulties were not recorded. TAT levels are highly affected by the duration of needle puncturing; the more time it takes to collect blood, the more the TAT levels increase [26]. This artifact can result in a pseudo-positive VTE prediction. It would have been better to recollect blood in case of blood sampling taking too long, with too high levels of the activation markers, or clotted blood. F1 + 2 levels are less affected by the duration of needle puncturing than TAT levels, however, TAT and F1 + 2 reflect slightly different results of coagulation activation. The combination of TAT and PIC might have a unique role for predicting VTE development.

## Conclusions

This study clearly showed that the prevalence and incidence of VTE were high in hospitalized Japanese patients receiving chemotherapy for malignancies, and suggests that non-increased PIC with increased TAT is an independent risk factor for VTE development. Activation markers of coagulation and fibrinolysis are easy to be tested, and may be useful to define the high-risk group. It is worth being investigated further with a large number of patients and including other activation markers.

## Abbreviations

CI: Confidence interval
CT: Computed tomography
DVT: Deep vein thrombosis
F1 + 2: Prothrombin fragment 1 + 2
OR: Odds ratio
PIC: Plasmin α2-plasmin inhibitor complex
TAT: Thrombin-antithrombin complex
VTE: Venous thromboembolism
